# Impact of Subinhibitory Concentrations of Metronidazole on Morphology, Motility, Biofilm Formation and Colonization of *Clostridioides difficile*

**DOI:** 10.3390/antibiotics11050624

**Published:** 2022-05-05

**Authors:** Tri-Hanh-Dung Doan, Marie-Françoise Bernet-Camard, Sandra Hoÿs, Claire Janoir, Séverine Péchiné

**Affiliations:** 1 UniCancer Group, 94270 Le Kremlin-Bicêtre, France; th-dungdoan@unicancer.fr; 2 Université Paris-Saclay, INRAE, AgroParisTech, Micalis Institute, 78350 Jouy-en-Josas, France; marie-francoise.bernet-camard@universite-paris-saclay.fr (M.-F.B.-C.); sandra.hoys@universite-paris-saclay.fr (S.H.); claire.janoir-jouveshomme@universite-paris-saclay.fr (C.J.)

**Keywords:** *Clostridioides difficile*, metronidazole, biofilm, motility, morphology, adherence, colonization

## Abstract

*Clostridioides difficile* infection (CDI) is the primary cause of health-care-associated infectious diarrhea. Treatment requires mostly specific antibiotics such as metronidazole (MTZ), vancomycin or fidaxomicin. However, approximately 20% of treated patients experience recurrences. Treatment with MTZ is complicated by reduced susceptibility to this molecule, which could result in high failure and recurrence rates. However, the mechanism remains unclear. In this study, we investigated the impact of subinhibitory concentrations of MTZ on morphology, motility, biofilm formation, bacterial adherence to the intestinal Caco-2/TC7 differentiated monolayers, and colonization in monoxenic and conventional mouse models of two *C. difficile* strains (VPI 10463 and CD17-146), showing different susceptibility profiles to MTZ. Our results revealed that in addition to the inhibition of motility and the downregulation of flagellar genes for both strains, sub-inhibitory concentrations of MTZ induced various in vitro phenotypes for the strain CD17-146 exhibiting a reduced susceptibility to this antibiotic: elongated morphology, enhanced biofilm production and increased adherence to Caco-2/TC7 cells. Weak doses of MTZ induced higher level of colonization in the conventional mouse model and a trend to thicker 3-D structures entrapping bacteria in monoxenic mouse model. Thus, sub-inhibitory concentrations of MTZ can have a wide range of physiological effects on bacteria, which may contribute to their persistence after treatment.

## 1. Introduction

*Clostridioides difficile* (CD) is a Gram-positive, spore forming and obligate anaerobic bacilli, responsible for various intestinal symptoms from mild diarrhea to severe pseudomembranous colitis and is the primary cause of antibiotic-associated diarrhea in developed countries [1].

For decades, vancomycin and MTZ were widely used as first-line therapy. However, the emergence and spread of *C. difficile* clinical isolates resistant to MTZ led to a recent update guideline to recommend MTZ only as an alternative to vancomycin or fidaxomicin for an initial non-severe *Clostridioides difficile* infection (CDI) treatment due to its high failure and high recurrence rates (20–25%) [2,3]. One possible factor that may explain MTZ treatment failure is pharmacokinetics of the antibiotic. Indeed, when MTZ is administered orally, at least 80 percent of the drug is absorbed in one hour. Fecal elimination and colonic (*C. difficile* infection site) concentrations are low [4]. In symptomatic patients, stool concentrations of MTZ were detected with a mean concentration of 9.3 µg/g in watery stool and 1.2 µg/g in formed stool. In asymptomatic patients, MTZ were undetectable [5]. The poor fecal concentrations of MTZ might result in insufficient antibiotic concentrations to inhibit vegetative bacteria, promoting the development of adaptation and resistance mechanisms of *C. difficile*.

Indeed, some studies indicate that MTZ resistance in *C. difficile* is heterogeneous, which means that growth in the presence of MTZ may select slow growing subclones with higher MTZ minimum inhibitory concentrations (MIC) from a population with low MIC [6]. The slowly growing or non-growing state of bacterial persisters is due to a general arrest in metabolic activity which is thought to give them the ability to survive exposure to antibiotics [7]. Moura et al. also found that following the exposure to subinhibitory concentrations of this antibiotic, *C. difficile* strains PCR ribotype 001 and 010 showed increased MIC [8]. MTZ heteroresistant *C. difficile* can obviously be a matter of concern, resulting in therapeutic failures.

In addition, the production of *C. difficile* biofilm is proved to be induced by the exposure to MTZ at subinhibitory concentrations [9]. In vitro, biofilm formation has been reported to be an important factor contributing to antimicrobial resistance of *C. difficile* by forming a multilayered structure encased in a matrix containing proteins, DNA, and polysaccharides [10]. In particular, cells of toxigenic *C. difficile* strains NAP1/027 R20291 grown in biofilm showed a 100-fold increase in the resistance to antibiotic compared to planktonic cells [10]. Higher biofilm formation could participate to better colonization and persistence in vivo and so far, persistent recurring *C. difficile* infections have been a major challenge in the treatment of CDI [11]. Indeed, recurrent bacterial infections occur with the ability to produce resilient biofilms by various pathogens [12].

Exposure to sub-MIC levels of antibiotics has been found to cause substantial increase in bacterial adherence to eukaryotic host cells and induced biofilm formation for several pathogenic species. Subinhibitory concentrations of ciprofloxacin were shown to increase bacterial adherence to host tissue by upregulating the expression of fibronectin-binding proteins in *S. aureus*. This increased expression involves two pathways: upregulation of the stress-response sigma factor SigB and induction of the SOS response (RecA and LexA) [13]. Moreover, in *P. aeruginosa*, aminoglycoside antibiotics have been shown to induce biofilm formation. This response requires a functional *arr* gene, which encodes an inner membrane phosphodiesterase, whose substrate is cyclic di-guanosine monophosphate (c-di-GMP), a second messenger that inhibits bacterial motility and promotes cell surface adherence and biofilm formation [14].

Thus, we thought of great interest to examine impacts of low doses of MTZ on the ability of *C. difficile* strains to form a biofilm and to colonize mice.

In this study, we compare characteristics (morphology, motility, in vitro bacterial adherence, and biofilm production) of two strains of *C. difficile*, the CD17-146 with reduced susceptibility to MTZ and the VPI 10463 sensitive to MTZ, in absence and presence of MTZ at subinhibitory concentrations. Besides, we also determine in vivo the effect of low doses of MTZ on the colonization process of these strains in a conventional mouse model. Finally, distributions of each strain over the cecal tissue in a mono-associated mouse model were visualized by confocal laser scanning microscopy.

## 2. Results

### 2.1. Impact of MTZ on the Morphology of C. difficile

**Figure 1:**
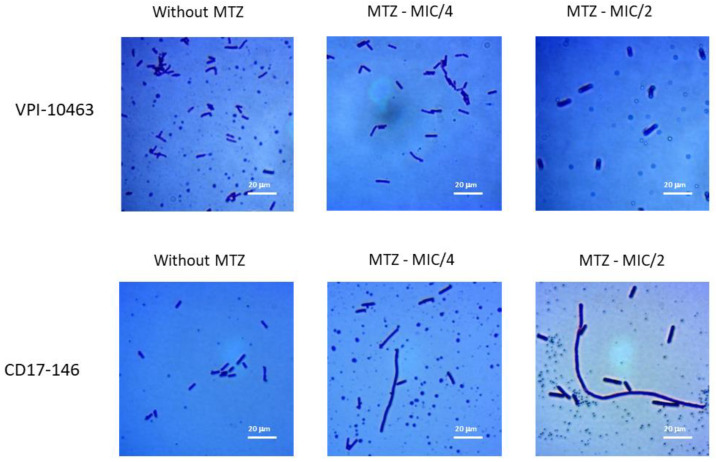
Morphology of bacterial cells at subinhibitory concentrations of MTZ. VPI 10463 and CD17-146 were grown in BHI-SG broth without MTZ or with MTZ at MIC/4 and MIC/2 until OD600 nm of cultures reached 0.4. Gram-stain images of CD17-146 at 100× magnification demonstrated an elongation of bacterial cells.

The VPI 10463 *C. difficile* strain, isolated from an abdominal wound, is Tcd A and Tcd B positive, and belongs to PCR-ribotype 087. The CD17-146 isolate is a non-toxigenic strain from PCR ribotype 596, displaying reduced susceptibility to MTZ. The MIC values for MTZ determined as described in Material and Method section, were 1 μg/mL for CD17-146 and 0.5 μg/mL for VPI 10463. Morphological analyses were performed on the untreated cultures (without MTZ) as well as cultures exposed to MTZ at concentration of MIC/4 and MIC/2. Morphological analyses were performed on the untreated cultures (without MTZ) as well as cultures exposed to MTZ at concentration of MIC/4 and MIC/2. Optical microscopic observation on Gram staining showed that CD17-146 strain was grown into filaments with subinhibitory concentrations of MTZ. On the contrary, this morphology change was not observed in VPI 10463 cultures treated with MTZ whatever the subinhibitory concentration used (Figure 1).

### 2.2. C. difficile Biofilm Production in Absence and Presence of MTZ

Biofilm formation has been reported to be induced by subinhibitory concentrations of MTZ in three *C. difficile* isolates belonging to PCR-ribotype 010 [9]. To study whether MTZ stimulates biofilm formation of VPI 10463 and CD17-146 strains in vitro, bacteria were grown in BHISG with a range of concentrations of MTZ (0 to 2 µg/mL) and biofilm formation was measured after 48 h by crystal violet staining and viable cell and spore enumeration.

**Figure 2:**
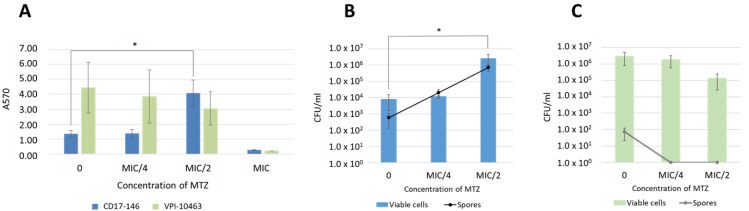
Biofilm quantification of VPI 10463 and CD17-146 at subinhibitory concentrations of MTZ. Bacteria were cultivated in BHI-SG broth without or with MTZ at MIC/4 and MIC/2 at 37 °C under anaerobic conditions to form biofilms. After 48 h, the biofilm mass was quantified by crystal violet staining. A 4-fold increase of biomass was observed in the CD17-146 strain at MIC/2 (**A**). Panels (**B**) and (**C**) depicts enumeration of vegetative forms and spores included in biofilms formed the two strains CD17-146 and VPI 10463, respectively. Data are means of at least three independent experiments, each performed in triplicate. The error bars represent standard deviation. Significantly different (*p* < 0.05) ratios are indicated by asterisks (Man-Whitney test).

In the absence of MTZ, a significant higher biofilm production was observed in VPI 10463 strain compared to CD17-146 strain. We found a declining trend of biomass and vegetative forms in VPI 10463 strain at MTZ subinhibitory concentrations (Figure 2A,C). However, differences observed were not statically significant. Interestingly, there were no spores in biofilm of VPI 10463 at MIC/4 and MIC/2. (A570 values were 4.44 ± 1.69 and 1.35 ± 0.22 in VPI 10463 and CD17-146, respectively). Differently, when MTZ was added, a significant 4-fold increase of biomass (A570 value 4.08 ± 0.87) was observed in the CD17-146 strain at MIC/2 (0.5 µg/mL) (Figure 2A), indicating a strong induction of biofilm formation in this strain by MTZ. In accordance with results of the quantitative biofilm assay by crystal violet, the viable cells and spores of this strain went up dramatically at MIC/2 with an increase of two log (Figure 2B).

These results highlight different behaviors of two strains in presence of MTZ.

### 2.3. Impact of MTZ on the Motility of C. difficile and Transcriptions of Flagellar Genes

**Figure 3:**
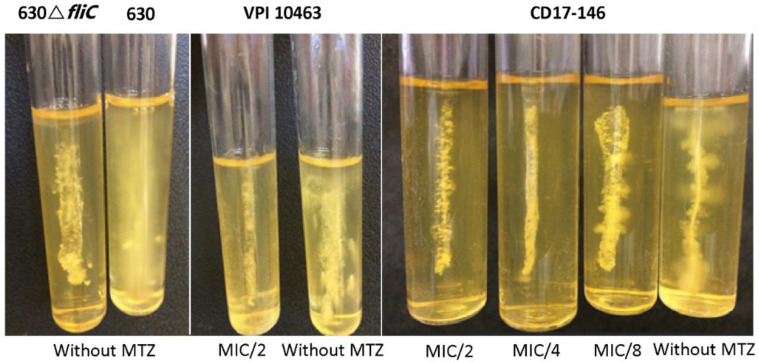
Motility of VPI 10463 (VPI) and CD17-146 (146) at subinhibitory concentrations of MTZ. Bacterial strains were inoculated in BHI medium containing 0.3% agar without or with MTZ at the concentrations 0.125, 0.25 or 0.5 µg/mL and grown anaerobically at 37 °C for 48 h. MTZ reduced the motility of both *C. difficile* strains through soft agar and this effect is concentration dependent.

As the motility and bacterial flagella are known to modulate attachment and biofilm production, we used a semi-soft agar assay to monitor the effect of MTZ on motility of *C. difficile*. We observed a significant decrease in the mobility of both *C. difficile* strains through soft agar and the effect is concentration dependent. The motility of *C. difficile* decreased with increasing concentrations of MTZ. This suggested that MTZ might impede transcription of flagellar genes (Figure 3). For this study, the flagellated 630 flagellated and the unflagellated 630∆*fliC* (deletion mutant for *fliC* gene resulting in lack of the flagellar filament production) strains were used as positive and negative controls for motility assay, respectively.

**Figure 4:**
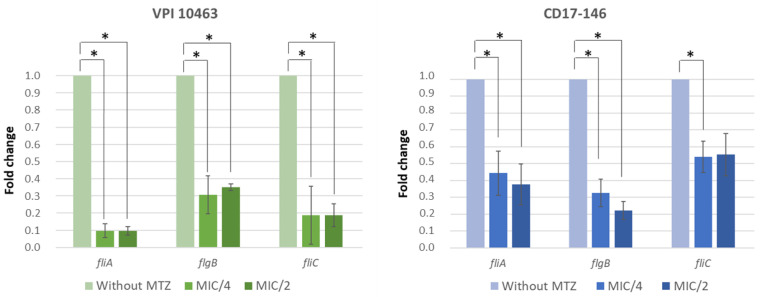
Expression of flagellar genes of VPI 10463 (VPI, green) and CD17-146 (146, blue) at subinhibitory concentrations of MTZ. Bacteria were grown in BHI-SG broth without or with MTZ at MIC/4 and MIC/2 at 37 °C under anaerobic conditions until OD600 nm reached 0.7. Using qRT-PCR, transcripts levels of *fliA*, *flgB* and *fliC* were measured. MTZ repressed the expression of flagellar genes in the two strains. Data are representative of three independent experiments, each performed in triplicate. The error bars represent standard errors of mean (SEM). Significantly different (*p* < 0.05) ratios are indicated by asterisks (Man-Whitney test).

To test this, *C. difficile* strains were cultured in the presence or absence of MTZ, and the levels of flagellar gene transcripts were measured by qRT-PCR. In both strains, subinhibitory concentrations of MTZ reduced the levels of fliC, flgB and fliA mRNAs, compared to the cultures without MTZ (Figure 4). The levels of transcription at MIC/4 were quite similar to the levels at MIC/2 for the two strains. For CD17-146 strain, the expression of *fliC* and *fliA* decreased around 2-fold, while *flgB* went down 3-fold at MIC/4 and 4-fold at MIC/2. For VPI 10463 the expression of these genes decreased more drastically: 10-fold, 3-fold and 5-fold for *fliA*, *flgB* and *fliC,* respectively. Furthermore, *gluD* (reference gene) transcript levels were equivalent in both strains grown with or without MTZ. Therefore, subinhibitory concentrations of MTZ had a global negative effect on transcription of flagellar genes.

### 2.4. Effect of Subinhibitory Concentrations of MTZ on C. difficile Adherence

Since the first step of infection is the colonization process which may include adherence to epithelial cells, we studied the impact of exposure to MTZ on *C. difficile* adherence to an intestinal cell Caco-2/TC7, a simple and human in vitro model.

**Figure 5:**
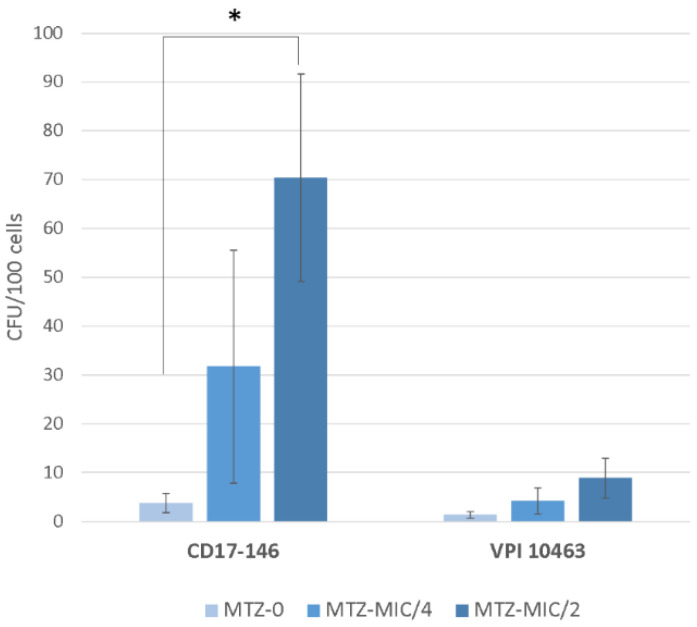
Impact of MTZ on adherence of VPI 10463 and CD17-146 strains to Caco-2TC7 cells. Overnight cultures of each *C. difficile* strain in BHISG broth, with or without subinhibitory concentrations of antibiotics, were pelleted and washed once with PBS, then incubated with Caco-2/TC7 cells in DMEM for 1 h 30 at 37 °C under anaerobic conditions. The adhesion ability was expressed as the number of adherent bacteria per 100 Caco-2/TC7 cells. The adherence to Caco-2/TC7 cells of CD17-146 exposure to MTZ at MIC/2 increased significantly. Data are representative of at least three independent experiments, each performed in triplicate. The error bars represent standard error of the mean (SEM). Significantly differences (*p* < 0.05) are indicated by asterisks (Man-Whitney test).

Counts of cell adherent bacteria showed that exposure to MTZ at MIC/2 increased significantly the adherence of CD17-146 to Caco-2/TC7 cells. On the contrary, there were no significant changes in the number of adherent bacteria observed in VPI 1043 whatever the sub-inhibitory concentrations of MTZ. Despite having the similar level of adherence without MTZ, the two strains responded differently to MTZ pressure (Figure 5).

### 2.5. Subinhibitory Concentrations of MTZ Stimulate Cecum Colonization by CD17-146 in the Conventional Mouse Model

**Figure 6:**
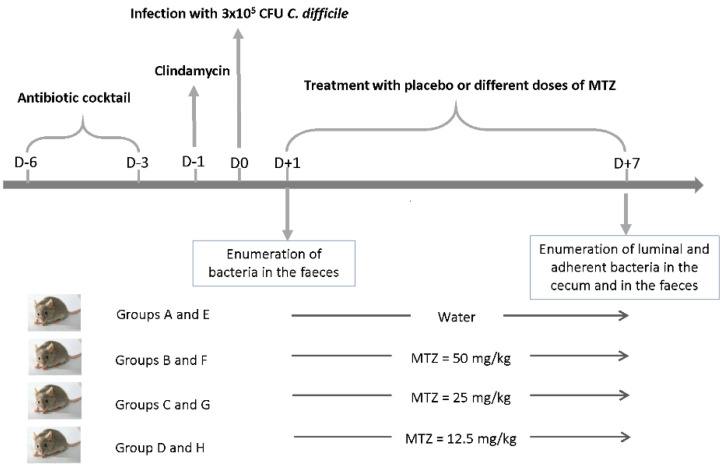
Experimental design of the conventional mouse model to study the impact of different doses of MTZ on the cecum colonization by VPI 10463 and CD17-146. The antibiotic cocktail contained kanamycin (40 mg/kg), gentamycin (3.5 mg/kg), colistin (8.5mg/kg), MTZ (21.5 mg/kg) and vancomycin (4.5 mg/kg). The clindamycin was administrated intraperitoneally (10 mg/kg). The mice were divided into 8 groups, 6 mice per group: four groups (A–D) infected with VPI-10463 and four groups (E–H) infected with CD17-146.

After having observed different impacts of MTZ on in vitro biofilm formation and on the adherence to a human intestinal cell model for the two *C. difficile* strains, we decided to study their colonization fitness in conventional mice receiving different regimen of MTZ. The experiment design is described in Figure 6.

The bacterial burden was quantified by seeding fecal (on days 1 and 7 post-infection) and cecal (on day 7 post-infection) samples on selective plates.

After infection, both strains proliferated and reached a bacterial burden of approximately 3 × 10^7^ bacteria per gram of feces after 24 h. As expected, mice infected by VPI 10463 showed signs of clinical illness with weight loss and severe diarrhea, especially from 48 h to 72 h post-challenge. Approximately 80% of mice died in group A non treated with MTZ and only two mice survived after 7 days. In group C treated with a half and D treated with a quarter of usual dose, mortality rates were about 30–40%. In contrast, there were no deaths in the group B treated with usual dose. On the other hand, mice infected with CD17-146 did not have any signs of illness, consistent with the non-toxigenic status of this strain.

**Figure 7:**
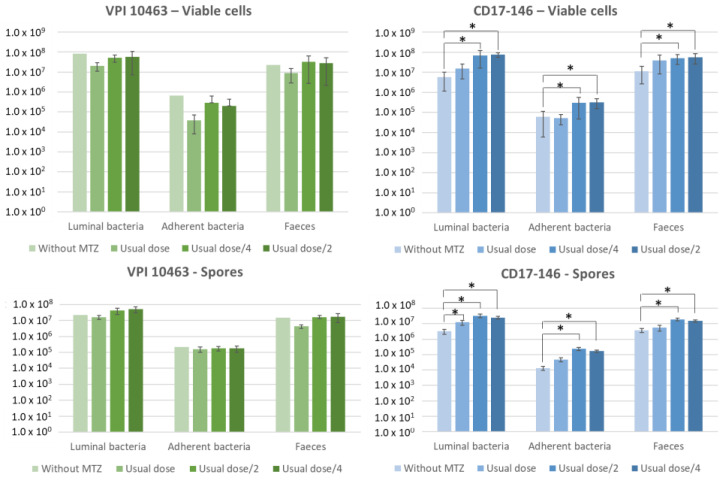
Impact of MTZ on cecum colonization of *C. difficile* in conventional mice, 7 days post-infection. Conventional mice were infected by either VPI 10463 (green) or CD17-146 (blue) with equivalent number of vegetative cells. Colonization process and bacterial burden were monitored by seeding fecal, cecal contents (luminal bacteria) and homogenized mucosal tissues (adherent bacteria) on selective plates at day 7 post-infection. Low doses (usual dose/4 and usual dose/2) of MTZ stimulated the colonization of CD17-146. Data generated from two independent experiments. The error bars represent standard error of the mean (SEM). Significant differences (*p* < 0.05) compared to group without treatment are indicated by asterisks (Man-Whitney test).

The levels of intestinal colonization reached by each strain at day 7 are shown in Figure 7. Overall, without MTZ, VPI 10463 strain significantly colonized better than CD17-146 (*p* = 0.025) but their rates of colonization were similar in presence of MTZ. Indeed, the treatment of MTZ did not impact the colonization of VPI 10463 strain: number of spores and vegetative cells in cecum and feces were similar between the non-treated group and groups treated with MTZ, even with the group treated with the highest dose of MTZ (50 mg/kg).

For the groups infected by CD17-146, we observed a 10-fold significant increase of bacterial burden in cecum (for both luminal and adherent bacteria) and in feces after treatment of MTZ with doses of 12.5 (group H) and 25 (group G) mg/kg. In contrast, there were no significant differences between the bacterial burden in mice treated with usual dose and non-treated. Our results suggest that subinhibitory concentrations of MTZ stimulated the mouse intestinal colonization by CD17-146 *C. difficile* strain.

### 2.6. Visualisation of Bacterial Distribution in the Cecum by CLSM

Our results in conventional mouse model showed that doses lower than the usual one of MTZ had the same impacts on the colonization of CD17-146 for usual dose/4 and usual dose/2. To study the spatial organization of the two strains in the cecum, we then chose the usual dose/4 (12.5 mg/kg) for visualization of bacterial distribution by confocal laser microscopy in a monoxenic mice model. One mouse in the group A infected by VPI 10463 and treated with water died 3 days post-infection. In the other groups, all three mice survived after 7 days.

The distribution of *C. difficile* in the cecum was heterogeneous. Irrespective of strain, we observed areas without and with bacteria associated with tissues. The cecal mucosa-associated bacteria were entrapped in a 3-D structure and displayed mainly isolated bacteria. (Figure S1).

**Figure 8:**
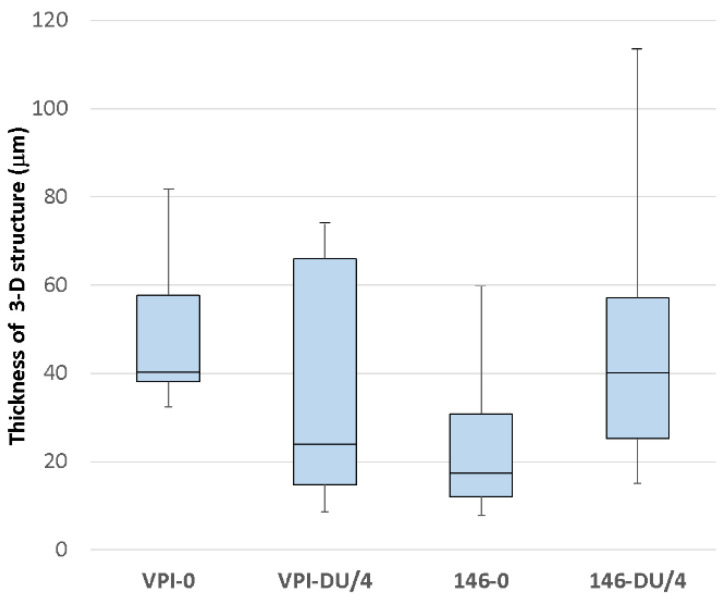
Thickness of bacterial 3-D structure of VPI 10463 (VPI) and CD17-146 (146) in cecum of mono-associated mouse model treated with placebo or with a quarter of usual dose of MTZ. The thickness of the bacterial 3-D structure is defined by the height on which bacteria are distributed. The thickness was determined directly from confocal Z-stack images. At least three mice were used for CLSM analyses for each strain, and at least 8 fields per sample were observed. Data are presented as boxplots with median and minimum-maximum whiskers. No significant difference was observed between strains (Mann-Whitney test).

We estimated the thickness of the bacterial layer present in tissues at different regions of the cecum. For VPI 10463 strain, without MTZ the thickness varies from 32.5 μm to 81.6 μm (average: 51.63 μm) and from 8.58 μm to 112.2 μm at a quart of usual dose of MTZ (average: 23.01 μm). The bacterial layer seemed to be thinner in presence of 12.5 mg/kg of MTZ but the difference was not significant due to a large variation (Figure 8). In contrast, the mean of thickness of CD17-146 strain had tendency to increase in the group treated with MTZ, from 25.71 μm without MTZ to 43.86 μm with MTZ. However, with this strain, we also found randomly distributed areas either with a high or a low thickness of the *C. difficile* community, from 7.73 μm to 59.73 μm in the group placebo and from 15.05 μm to 113.4 μm in the group treated with 12.5 mg/kg of MTZ. Therefore, there were no statistically significant difference in mean thickness between two groups (Figure 8).

The levels of intestinal colonization by each strain at day 7 are showed in supplemented data (Figure S2). In this model, no difference in intestinal colonization was observed neither between the two strains for the ability of colonize, nor between the strains in groups treated or not by MTZ.

## 3. Discussion

The increased antibiotic resistance reported for *C. difficile* clinical isolates and the recurrences of CDI are challenges facing physicians in the treatment of *C. difficile* infection. It has been estimated that approximately 27.3% of CDI treatment failures, as well as 23% of recurrences, are associated to treatment with MTZ [3].

As previously mentioned, the mechanisms of reduced susceptibility to MTZ are complex. Data obtained in recent studies on PCR-ribotype 027 and RT010 strains suggest that the reduced susceptibility is a multifactorial process involving alterations in different metabolic pathways, such as nitroreductase activity, iron uptake, and DNA repair [8,15]. Interestingly, a recent study showed the correlation between resistance to MTZ (MIC = 8 µg·mL^−1^) and the presence of a plasmid, pCD-METRO, in toxigenic and non-toxigenic strains. One of the plasmidic open reading frames (ORFs) showed homology at the protein level to the *nimB* gene of 5-nitroimidazole reductase described in *Bacteroides fragilis* [16]. Nitroimidazole reductase activity encoded by *nim* genes is supposed to reduce the nitro group of 5-nitroimidazole to an amino group leading to an inactivation of the compound [17]. Another study on chromosomal resistance to MTZ in *C. difficile* demonstrated truncation of the ferrous iron transporter FeoB1 could result in a low-level resistance. Higher-level resistance could be achieved by sequential acquisition of mutations in catalytic domains of pyruvate-ferredoxin/flavodoxin oxidoreductase, a synonymous codon changes to putative xanthine dehydrogenase, and frameshift and point mutations that inactivated the iron-sulfur cluster regulator (IscR). However, resistance involving these genes was observed only in the *feoB1* deletion mutant and not in the isogenic wild-type parent [18]. To go on further the comprehension of the mechanism involved in the bacterial response to MTZ, it could be interesting to study more precisely the nitroreductase or iron transport activities of these two strains, CD17-146 and VPI 10463.

Occasional filamentous forms, accompanied by generalized defects in MTZ transport, have been described in MTZ-resistant mutant of *B. fragilis* [19]. Likewise, *Escherichia coli* that survived high concentration MTZ challenge exhibited an elongated filamentous morphology [20]. Sublethal MTZ concentration also induced elongation of *Fusobacterium nucleatum* and *Porphyromonas gingivalis* [20,21]. In accordance with previous study, we observed a cell elongation in this study with CD17-146 strain with reduced susceptibility to MTZ. Overall, an elongated morphology was associated with MTZ reduced susceptibility in different bacteria. Changes in morphology suggests modifications in cell wall structure which may result in decreased MTZ uptake, one parameter involved in resistance to this drug [22].

Moreover, antibiotic pressure has been shown to enhance biofilm formation in different bacterial species, including *C. difficile* [10,12,23,24]. In accordance with previous studies, we have shown a significant increase of in vitro biofilm formation in strain CD17-146 with reduced susceptibility in presence of MTZ at MIC/2. On the contrary, the ability of the susceptible strain VPI 10463 to produce biofilm in the same conditions did not change when MTZ was added to culture medium. Without MTZ, VPI 10463 was a strong biofilm-producer compared to CD17-146. A previous study has also demonstrated, in the presence of MTZ, a significant increase in biofilm formation in moderate-biofilm forming bacteria, not observable in strongly biofilm-forming strain [9]. Furthermore, Rahmoun et al. compared different susceptible isolates, and a statistically higher percentage of isolates with reduced susceptibility to metronidazole or vancomycin were shown to be biofilm producers [25].

Bacterial flagella are known to influence the adherence step in biofilm formation in motile bacteria. According to our results on biofilm formation, MTZ demonstrated a concentration-dependant inhibition effect on the expression of some flagellar genes (*fliC*, *flgB*, *fliA*) and the motility in both strains. A downregulation of flagellar genes leading to an impaired motility may be a factor for the increased MTZ-induced biofilm production in CD17-146. Differently, a decrease in the expression of flagellar genes and motility by antibiotic pressure did not further increase biofilm production in strain VPI 10463. Previous researches indicate that the precise role of flagella varies between strains. Indeed, strain 630 *C. difficile fliC* and *fliD* mutants were reported to have better adherence on Caco-2 cells, suggesting that flagella and motility may interfere with *C. difficile* adherence to epithelial cell surfaces [26]. In contrast, all flagella mutants (*fliC*, *fliD* and *flgE*) of the epidemic strain R20291 were less effective in adherence to Caco-2 cells than the wild-typein [27].

Our results on Caco-2/TC7 cells- showed that bacterial adherence increased by MTZ at MIC/2 for the CD17-146 strain. As the concentrations of MTZ in watery stools following oral therapy range between 0.8 and 24.2 µg/g [5], it is possible that low concentrations of this antibiotic are present in the gut in some phases of CDI treatment (particularly at the beginning and the end) and that they could stimulate the adherence of *C. difficile* to gut epithelial cell, the first step of colonization. Indeed, our findings demonstrated that weak doses (under doses used usually in the mouse model) of MTZ increased the colonization of CD17-146 strain in cecum of conventional mice, especially the amount of bacteria associated with the cecal mucosa. The same result was not found for strain VPI 10463, which was shown to colonize better than CD17-146 in the cecum of mice, in absence of MTZ. We hypothesized that this could be due to the production of toxins by VPI 10463 while CD17-146 is non-toxigenic. Indeed, a previous study showed that sub-lethal concentrations of *C. difficile* TcdA was able to cause redistribution of plasma membrane components between distinct surface domains and facilitation of bacterial access to BL receptors, leading to a successful colonization of the colonic mucosa [28]. It is also worth noting that there were no significant differences in the level of colonization between the groups treated with usual dose of MTZ and the groups treated with water although MTZ significantly increased the survival rates. This result is in accordance with a previous study on *C. difficile* infection treatments in mice which indicated that MTZ did not reduced the number of spores in feces compared to the infected control group [29].

For several pathogenic bacteria, primary colonization and persistence in the host has been correlated with biofilm formation [30,31,32]. In addition, we observed in vitro that sub-inhibitory concentration of MTZ induced biofilm formation of CD17-146. We wondered if there was a link between the increased colonization of this strains and its biofilm production. Therefore, we visualized bacterial distribution in the cecum of monoxenic mice by CLSM. In monoxenic mice infected with CD17-146 strain, the cecal mucosa-associated bacteria formed a 3-D structure, as observed by CLSM analysis. Furthermore, we observed that the median thickness of these structures is increased when mice were treated at a quarter of usual dose of MTZ, although not in a significant manner, suggesting that low doses of MTZ may play a role in the enhancement of persistent structures by strain CD17-146 in this model, but further experiments should be done to confirm this hypothesis. This phenomenon was not observed for the VPI 10463 strain. The persistence of bacterial cells in the human intestine as a protective barrier provided by biofilm could have an important clinical relevance in the treatment failure and/or recurrence of infections associated with *C. difficile* strains. Indeed, *C. difficile* cells in biofilms show specific features that may facilitate the infection, such as spore formation and toxins A and/or B production [33,34] and resistance to antibiotics [10].

Overall, the two strains responded differently to the stress induced by MTZ subinhibitory concentrations except for the decreased motility which occurred in both strains. For CD17-146, strain with reduced susceptibility to MTZ and a moderate biofilm-forming ability without MTZ, we observed under low MTZ concentrations an elongation morphology, increased biofilm production and higher level of colonization in conventional mice and a trend to thicker 3-D bacterial structures at the surface of the cecal mucosa. On the other hand, for VPI 10463, a MTZ sensitive and strong biofilm-forming strain, we did not observe these changes under MTZ pressure. More investigations are now necessary to unravel the different aspects of this complex mechanism.

Our previous proteomic analyzes suggested that the increase of biofilm production could be related to the decrease in production of the protease Cwp84, a cell wall protein, and a higher production of an aminotransferase of the MocR family [35]. Indeed, we previously observed in presence of MTZ at MIC/2 a 3-fold decrease in the amount of Cwp84 in CD17-146. Cwp84 protease cleaves the S-layer protein SlpA on bacterial surface into two subunits. The *cwp84* mutant strain was shown to grow slower and elaborated more robust biofilms compared with the parental *C. difficile* 630Δerm strain. Furthermore, bacterial load of mutant strain in vivo competition assays was maintained over time in the cecum, suggesting there may be stable reservoirs of bacteria and these reservoirs may ultimately transition into the biofilm state [36]. Proteomic analyzes also revealed a 3-fold increased amount of a putative aminotransferase for CD17-146 strain at MIC/2. This protein belonging to MocR family 2 shares 27% identity with PdxR of *Streptococcus mutans*. Interestingly, PdxR is known to have a role in biofilm formation of S. mutans since the pdxR mutant forms significantly fewer biofilm compared to its parental strain [37]. Further research is required to elucidate the mechanism of biofilm induction in CD17-146 strain by MTZ.

Finally, we have mentioned several hypotheses that could explain the greater bacterial persistence with certain strains of *C. difficile*. However, additional experiments should be considered in order to elucidate the exact mechanism involved in this phenomenon. We are aware that these findings on the impact of MTZ on colonization by *C. difficile* were obtained from a limited number of strains and therefore need to be extended to a larger panel of a variety of strains to confirm the relevance of our results to other clinical situations. However, to our knowledge, this report is the first description of the effect of low dose of MTZ on the colonization and cecal distribution of *C. difficile* in vivo.

## 4. Materials and Methods

### 4.1. Bacterial Strains and Antibiotic Susceptibility

Two *C. difficile* strains VPI 10463 and CD17-146 were used in this study. The CD17-146 isolate provided by the *C. difficile* French National Center in Saint Antoine hospital (Paris, France) was stored immediately after isolation at −80 °C. This strain has been shown to be a non-toxigenic strain and belonging to the PCR ribotype 596 with reduced susceptibility to MTZ (minimum inhibitory concentration determined by ETEST^®^ on solid agar was 2 µg/mL). The MIC values for MTZ evaluated by broth dilution method in our laboratory were 1 μg/mL for CD17-146 and 0.5 μg/mL for VPI 10463. According to epidemiological cut-off values of the European Committee on Antimicrobial Susceptibility Testing (ECOFF EUCAST2015), resistance to MTZ was defined as MIC > 2μg/mL. Most *C. difficile* susceptible strains have MIC ≤ 0.5 μg/mL (https://mic.eucast.org/Eucast2/regShow.jsp?Id=21294, accessed date: 15 January 2021). Thus, we considered CD17-146 as a strain with reduced susceptibility.

Bacteria were grown at 37 °C under anaerobic conditions (90% N_2_, 5% CO_2_ and 5% H_2_).

### 4.2. Morphology Observation

Bacteria were grown in BHISG (Brain Heart infusion broth, Difco, Detroit, MI, USA, supplemented with 1.8% Glucose, 0.1% L-Cysteine and 0.5% yeast extract) under subinhibitory concentrations (MIC/4 and MIC/2 for each strain) of MTZ, at 37 °C under anaerobic conditions. When OD600 nm of cultures reaches 0.4, bacteria were observed by optical microscopy after Gram staining.

### 4.3. In Vitro Biofilm

Biofilm assays were performed in 24-well polystyrene plates (Costar, Washington, DC, USA). Overnight cultures of each *C. difficile* strain in BHISG broth were diluted in fresh BHISG to obtain OD600 nm = 0.05 and 1 mL of diluted pre-culture was added to each well. Plates were incubated at 37 °C under anaerobic conditions for 1 h and the medium was removed to eliminate non-adherent cells. Then, 900 µL of BHISG and 100 µL of a solution of MTZ were added to each well to obtain final concentrations of 0.125, 0.25, 0.5, 1 and 2 µg/mL. For control wells, 100 µL of sterile water was added instead of MTZ. After 48 h of incubation, the supernatant was removed carefully, and wells were gently washed twice with sterile phosphate-buffered saline (PBS). The biofilm was thereafter quantified by crystal violet staining (ACROS OrganicsTM. Somerville, NJ, USA) as previously described [10,38], and by enumeration of viable cells and spores. For viable cell enumeration, 1 mL of sterile pre-reduced PBS was added after washing step to each well, the biofilm formed in the bottom was scraped, the suspension was then diluted and plated on BHI agar supplemented with 3% defibrinated horse blood. For spores, the suspension of biofilm was treated with ethanol 96% in the proportion 1:1 one hour before the enumeration on BHI agar supplemented with 3% defibrinated horse blood and 0.1% taurocholate. The assay was performed in triplicate. Two-tailed, Mann Whitney test with SPSS 20 software was used to evaluate whether the differences observed in the presence or absence of antibiotic were significant for each strain. Differences were considered statistically significant for *p* values < 0.05.

### 4.4. Motility Assays

Motility assays were performed using motility agar tubes containing BHI (Brain Heart infusion broth, Difco, USA) medium and 0.3% agar with MTZ at final concentrations of 0.125, 0.25, 0.5 µg/mL. These were stab inoculated and grown anaerobically at 37 °C for 48 h [39]. Control cultures contained no antibiotics. The motile strains *C. difficile* 630 and non-motile mutant strains *C. difficile* 630 ∆fliC were used as control [27].

### 4.5. RNA Extraction and Quantitative RT-PCR Analysis on Flagellar Genes

*C. difficile* cultures were grown to an OD600 nm of 0.7 in BHISG, without or with MTZ at MIC/4 or MIC/2, RNAs were extracted using Trizol Reagent (Thermo Fisher Scientific, Waltham, MA, USA). cDNA was synthesized from 1 µg RNA using random primers and SuperScript™ III Reverse Transcriptase (Invitrogen, Waltham, MA, USA) as described by the manufacturer. Real-time PCRs were done with 1 ng of cDNA template using SSo Advanced™ SYBR Green Supermix (Bio-Rad, Hercules, CA, USA). The primers used for the three genes detected *fliC*, *flgB* and *fliA* are listed in Supplementary Table S1, *fliC* coding flagellin in the F1 region of flagellar operon; f*lgB*, which is located at the beginning of F3 region; and *fliA*, located near the end of the F3 region which encodes a sigma factor predicted to activate flagellar gene expression in F1 [40]. Reactions were run on a CFX96 Real-time system (Bio-Rad) with the following cycling conditions: 30 s polymerase activation at 95 °C and 40 cycles at 95 °C for 5 s and 60 °C for 10 s. In order to verify the specificity of the real-time PCR reaction for each primer pair, an additional step from a start at 65 °C to 95 °C (0.5 °C/0.5 s) was performed to establish a melting curve. The *gluD* gene was used as reference gene, as described previously [41]. Normalized relative quantities were calculated using the ΔΔCT method. Data were analyzed with Student t test with SPSS 20. Results are expressed as mean ± standard error of mean (SEM).

### 4.6. Adherence Assays

The enterocyte-like Caco-2/TC7 cell line was used between passages 25 and 35. Cells were grown in DMEM medium (Dulbecco’s modified Eagle’s minimum, Gibco, Waltham, MA, USA) supplemented with 15% fetal calf serum (Gibco, United States) and 1% non-essential amino acids NEAA (Gibco, USA). The Caco-2/TC7 monolayers were inoculated in 24-well polystyrene culture plates (TPP, Dominique Dutscher SAS, Brumath, France) with 25,000 cells per well and used 14 days after seeding, when cells were differentiated [42].

Prior to adherence assays, cells were washed twice with PBS and 0.5 mL DMEM was added to each well. Overnight cultures of each *C. difficile* strain in BHISG broth, supplemented with subinhibitory concentrations of MTZ, were pelleted (2500 rpm, 5 min) and washed once with PBS. Then, 5 × 10^7^ CFU in 0.5 mL DMEM were added to each well. Bacteria and cells were incubated together for 1 h 30 at 37 °C under anaerobic conditions and wells were washed twice with PBS to discard non-adherent bacteria. After, cells were lysed with 1 mL of 1% saponin per well during 15 min at 4 °C and appropriate dilutions were spread on BHI agar plates supplemented with 3% horse blood (bioMérieux) for enumeration of cell-adherent vegetative bacteria. Bacterial colonies were counted after 48 h of incubation and results were expressed as CFU of cell-adherent bacteria per 100 cells. Assays were carried out in triplicate in three separate experiments. Data were analyzed with Mann-Whitney U test with SPSS 20. Results are expressed as mean ± standard error of mean (SEM).

### 4.7. Model of Conventional Mice to Study the Impact of MTZ on the Intestinal Colonization of C. difficile

The model used was based on the model developed in previous studies [29,43]. Figure 6 previously presented illustrates the experimental scheme. All animal experiments were performed according to European Union guidelines for the handling of laboratory animals and all procedures were approved by the Ethics Committee CAPSUD (Protocol APAFIS#4617-2016032118119771 vI).

Six to eight weeks-old C57BL/6JOlaHsd female mice, with an initial bodyweight of 16–19 g, were obtained from Charles River. Mice were grouped by 3 animals per cage in ventilated isolators and fed with autoclaved standard chow and water ad libitum throughout the experiment.

In order to disrupt the normal enteric microbiota and established *C. difficile* infection, mice were pretreated with an antibiotic mixture containing kanamycin (40 mg/kg), gentamycin (3.5 mg/kg), colistin (85,000 mg/kg), MTZ (21.5 mg/kg) and vancomycin (4.5 mg/kg). This cocktail was administered from day 6 to day 3 before infection in the drinking water. The concentrations of antibiotics were calculated based on the average weight of the mice and their expected water consumption. Then, mice were switched back to regular drinking water. One day prior to infection, mice received a single dose of clindamycin (10 mg/kg) by intraperitoneal route. On day 0, mice were challenged by oral gavage with approximately 3 × 10^5^ vegetative cells in 0.3 mL volume. This inoculum was prepared as follows: an overnight culture in BHISG was diluted in BHISG to a final concentration of approximately 1 × 10^5^ vegetative cells per mL, estimated by microscopic cell counting. The bacterial concentration was checked thereafter by enumerating vegetative viable cells. The treatments with MTZ began from 1-day post-infection (Figure 6) by oral gavage with different dose regimes. The mice were divided into 8 groups, 6 mice per group: four groups (A–D) infected with VPI 10463 and four groups (E–H) infected with CD17-146. Two groups (A and E) were treated with placebo (sterile water), six groups were treated with either usual dose of MTZ (B and F, 50 mg/kg), a half (C and G, 25 mg/kg) or a quarter (D and H, 12.5 mg/kg) of usual dose of MTZ twice a day, for 7 days. The usual dose defined in this study was the one used before to treat CDI in a mouse model [29].

Fecal samples were collected from each mouse on day 1 for enumeration of vegetative cells and were processed as previously described [44]. At the end of the 7-day observation period, mice were sacrificed, and the cecum and fecal samples were collected for enumeration of bacteria to assess the colonization rate in the cecum. The cecum contents were collected and used for luminal bacterial count. After three PBS rinses, the mucosal tissues were homogenized for 1 min with Ultra-Turrax T25 (IKA^®^, Labortechnik, Germany), and tissue-adherent bacteria were enumerated. Both vegetative cells and spores were enumerated in all samples. Vegetative cells were counted by plating serial 10-fold dilutions onto selective cycloserine-cefoxitin blood agar plates (CLO agar; bioMérieux, Marcy l’Etoile, France). Then, samples were mixed with equal volume of 96% alcohol for 1 h and spores were counted as described above. Colonies were counted after incubation anaerobically at 37 °C for 48 h.

Data were collected from two independent experiments and differences between two groups were analyzed with Mann-Whitney U test with SPSS 20. Results are expressed as mean ± standard error of mean (SEM).

### 4.8. Monoxenic Mouse Model to Visualize C. difficile Intestinal Distribution in the Cecum When Exposed to MTZ

#### 4.8.1. Animal Model

To visualize bacterial distribution over epithelial tissues in the cecum, we used the germ-free mouse model described by Soavelomandrosso et al. [38]. Six to eight weeks old germ-free C3H/HeN female mice were obtained from INRAE (Jouy-en-Josas, France). All animal experiments were performed according to European Union guidelines for the handling of laboratory animals and all procedures were approved by the Ethics Committee CAPSUD (Protocol APAFIS#23414-2019121910116284 v4).

Mice were housed in sterile isolators with ad libitum access to food and water. Before experiments, we checked the germ-free status of each animal as previously described [38]. Mice were challenged by oral gavage with 5 × 10^5^ CFU of *C. difficile*, either VPI 10463 (group A and B) or CD17-146 (group C and D) strain, with inoculum prepared as described for the conventional model. From 1-day post-infection, mice were treated with sterile water (group A and C) or with a quart of usual dose of MTZ: 12.5 mg/kg (group B and D) for 7 days by oral gavage twice a day. We used 3 mice per group. Seven days post-infection, feces were sampled for enumeration of bacteria and mice were euthanized. Data were analyzed with Mann-Whitney U test with SPSS 20. Results are expressed as mean ± standard error of mean (SEM). The caeca were collected for confocal microscopy analyses.

#### 4.8.2. Confocal Laser Scanning Microscopy (CLSM)

The spatial distribution of tissue-associated bacteria was determined by CLSM analysis of mouse mucosa from three mice for each strain. After removal of cecal content, the tissues were washed 3 times in 10 mL of PBS, spread on a glass slide and stained with the LIVE/DEAD^®^ BacLightTM 193 Bacterial Viability Kit (Thermo Fisher Scientific, United States): 20 µL of the diluted mixture (1:1000) was added on the tissues. Samples were visualized with a LSM 510 microscope (Carl Zeiss Inc., Oberkochen Germany). Horizontal plan images were acquired at several different locations for each sample. During the Z overlay, an average of the thickness of the bacterial layer in several places on the tissue sample was calculated. Finally, three-dimensional projections were reconstructed from x-z stacks using Imaris software (Bitplane, Belfast, UK).

## Supplementary Materials

The following supporting information can be downloaded at: https://www.mdpi.com/article/10.3390/antibiotics11050624/s1, Figure S1: Heterogeneous distribution of C. difficile over the cecal tissue in a mono-associated mouse model. Confocal laser-scanning microscopy 3-D projection of tissue-associated bacteria obtained from cecum for the CD17-146 without or with treatment of MTZ at 0.125 mg/kg, and the VPI 10463 without or with treatment of MTZ 0.125 mg/kg. Live cells (bacterial [rod] or epithelial) are labeled in green, dead cells are labeled in red. Scale bars (white): 30 μm. Figure S2: Impact of MTZ on cecum colonization of *C. difficile* in monoxenic mice, 7 days post-infection. Germ-free mice were infected by either VPI 10463 (group A and B) or CD17-146 (group C and D) strain with 5 × 10^5^ CFU of *C. difficile*. From 1-day post-infection, mice were treated with sterile water (group A and C) or with a quart of usual dose of MTZ: 12.5 mg/kg (group B and D) for 7 days by oral gavage twice a day. C. difficile shedding was monitored in feces at day 7 post-infection. There were no significant differences in colonization between the group treated with MTZ at usual dose/4 and the group non-treated for both strains. The error bars represent standard error of the mean (SEM). Table S1: Sequences of oligonucleotide primers used in this study.

## References

[1] De Roo A.C., Regenbogen S.E. (2020). *Clostridium difficile* Infection: An Epidemiology Update. Clin. Colon Rectal Surg..

[2] McDonald L.C., Gerding D.N., Johnson S., Bakken J.S., Carroll K.C., Coffin S.E., Dubberke E.R., Garey K.W., Gould C.V., Kelly C. (2018). Clinical Practice Guidelines for *Clostridium difficile* Infection in Adults and Children: 2017 Update by the Infectious Diseases Society of America (IDSA) and Society for Healthcare Epidemiology of America (SHEA). Clin. Infect. Dis..

[3] Johnson S., Louie T.J., Gerding D.N., Cornely O., Chasan-Taber S., Fitts D., Gelone S.P., Broom C., Davidson D.M. (2014). Polymer Alternative for CDI Treatment (PACT) investigators Vancomycin, Metronidazole, or Tolevamer for *Clostridium difficile* Infection: Results from Two Multinational, Randomized, Controlled Trials. Clin. Infect. Dis..

[4] Lau A.H., Lam N.P., Piscitelli S.C., Wilkes L., Danziger L.H. (1992). Clinical Pharmacokinetics of Metronidazole and Other Nitroimidazole Anti-Infectives. Clin. Pharmacokinet..

[5] Bolton R.P., Culshaw A.M. (1986). Faecal Metronidazole Concentrations during Oral and Intravenous Therapy for Antibiotic Associated Colitis Due to Clostridium Difficile. Gut.

[6] Peláez T., Cercenado E., Alcalá L., Marín M., Martín-López A., Martínez-Alarcón J., Catalán P., Sánchez-Somolinos M., Bouza E. (2008). Metronidazole Resistance in *Clostridium difficile* Is Heterogeneous. J. Clin. Microbiol..

[7] Andersson D.I., Hughes D. (2014). Microbiological Effects of Sublethal Levels of Antibiotics. Nat. Rev. Microbiol..

[8] Moura I.B., Spigaglia P., Barbanti F., Mastrantonio P. (2013). Analysis of Metronidazole Susceptibility in Different *Clostridium difficile* PCR Ribotypes. J. Antimicrob. Chemother..

[9] Vuotto C., Moura I., Barbanti F., Donelli G., Spigaglia P. (2016). Subinhibitory Concentrations of Metronidazole Increase Biofilm Formation in *Clostridium difficile* Strains. Pathog. Dis..

[10] Ðapa T., Dapa T., Leuzzi R., Ng Y.K., Baban S.T., Adamo R., Kuehne S.A., Scarselli M., Minton N.P., Serruto D. (2013). Multiple Factors Modulate Biofilm Formation by the Anaerobic Pathogen *Clostridium difficile*. J. Bacteriol..

[11] Surawicz C.M., Alexander J. (2011). Treatment of Refractory and Recurrent *Clostridium difficile* Infection. Nat. Rev. Gastroenterol. Hepatol..

[12] Hall-Stoodley L., Stoodley P. (2009). Evolving Concepts in Biofilm Infections. Cell. Microbiol..

[13] Li D., Renzoni A., Estoppey T., Bisognano C., Francois P., Kelley W.L., Lew D.P., Schrenzel J., Vaudaux P. (2005). Induction of Fibronectin Adhesins in Quinolone-Resistant *Staphylococcus aureus* by Subinhibitory Levels of Ciprofloxacin or by Sigma B Transcription Factor Activity Is Mediated by Two Separate Pathways. Antimicrob. Agents Chemother..

[14] Hoffman L.R., D’Argenio D.A., MacCoss M.J., Zhang Z., Jones R.A., Miller S.I. (2005). Aminoglycoside Antibiotics Induce Bacterial Biofilm Formation. Nature.

[15] Moura I., Monot M., Tani C., Spigaglia P., Barbanti F., Norais N., Dupuy B., Bouza E., Mastrantonio P. (2014). Multidisciplinary Analysis of a Nontoxigenic *Clostridium difficile* Strain with Stable Resistance to Metronidazole. Antimicrob. Agents Chemother..

[16] Boekhoud I.M., Hornung B.V.H., Sevilla E., Harmanus C., Bos-Sanders I.M.J.G., Terveer E.M., Bolea R., Corver J., Kuijper E.J., Smits W.K. (2020). Plasmid-Mediated Metronidazole Resistance in *Clostridioides difficile*. Nat. Commun..

[17] Alauzet C., Lozniewski A., Marchandin H. (2019). Metronidazole Resistance and Nim Genes in Anaerobes: A Review. Anaerobe.

[18] Deshpande A., Wu X., Huo W., Palmer K.L., Hurdle J.G. (2020). Chromosomal Resistance to Metronidazole in *Clostridioides difficile* Can Be Mediated by Epistasis between Iron Homeostasis and Oxidoreductases. Antimicrob. Agents Chemother..

[19] Britz M.L., Wilkinson R.G. (1979). Isolation and Properties of Metronidazole-Resistant Mutants of *Bacteroides fragilis*. Antimicrob. Agents Chemother..

[20] Jackson D., Salem A., Coombs G.H. (1984). The In-Vitro Activity of Metronidazole against Strains of *Escherichia coli* with Impaired DNA Repair Systems. J. Antimicrob. Chemother..

[21] Tally F.P., Sutter V.L., Finegold S.M. (1975). Treatment of Anaerobic Infections with Metronidazole. Antimicrob. Agents Chemother..

[22] Kwon Y.W., Lee S.Y. (2014). Effect of Sub-Minimal Inhibitory Concentration Antibiotics on Morphology of Periodontal Pathogens. Int. J. Oral. Biol..

[23] Bedran T.B.L., Grignon L., Spolidorio D.P., Grenier D. (2014). Subinhibitory Concentrations of Triclosan Promote *Streptococcus mutans* Biofilm Formation and Adherence to Oral Epithelial Cells. PLoS ONE.

[24] Wu S., Li X., Gunawardana M., Maguire K., Guerrero-Given D., Schaudinn C., Wang C., Baum M.M., Webster P. (2014). Beta- Lactam Antibiotics Stimulate Biofilm Formation in Non-Typeable *Haemophilus influenzae* by Up-Regulating Carbohydrate Metabolism. PLoS ONE.

[25] Abu Rahmoun L., Azrad M., Peretz A. (2021). Antibiotic Resistance and Biofilm Production Capacity in *Clostridioides difficile*. Front. Cell. Infect. Microbiol..

[26] Dingle T.C., Mulvey G.L., Armstrong G.D. (2011). Mutagenic Analysis of the *Clostridium difficile* Flagellar Proteins, FliC and FliD, and Their Contribution to Virulence in Hamsters. Infect. Immun..

[27] Baban S.T., Kuehne S.A., Barketi-Klai A., Cartman S.T., Kelly M.L., Hardie K.R., Kansau I., Collignon A., Minton N.P. (2013). The Role of Flagella in *Clostridium difficile* Pathogenesis: Comparison between a Non-Epidemic and an Epidemic Strain. PLoS ONE.

[28] Kasendra M., Barrile R., Leuzzi R., Soriani M. (2014). *Clostridium difficile* Toxins Facilitate Bacterial Colonization by Modulating the Fence and Gate Function of Colonic Epithelium. J. Infect. Dis..

[29] Erikstrup L.T., Aarup M., Hagemann-Madsen R., Dagnaes-Hansen F., Kristensen B., Olsen K.E.P., Fuursted K. (2015). Treatment of *Clostridium difficile* Infection in Mice with Vancomycin Alone Is as Effective as Treatment with Vancomycin and Metronidazole in Combination. BMJ Open Gastroenterol..

[30] Cooper R., Bjarnsholt T., Alhede M. (2014). Biofilms in Wounds: A Review of Present Knowledge. J. Wound Care.

[31] Mihai M.M., Holban A.M., Giurcaneanu C., Popa L.G., Oanea R.M., Lazar V., Chifiriuc M.C., Popa M., Popa M.I. (2015). Microbial Biofilms: Impact on the Pathogenesis of Periodontitis, Cystic Fibrosis, Chronic Wounds and Medical Device-Related Infections. Curr. Top. Med. Chem..

[32] Lund-Palau H., Turnbull A.R., Bush A., Bardin E., Cameron L., Soren O., Wierre-Gore N., Alton E.W.F.W., Bundy J.G., Connett G. (2016). *Pseudomonas aeruginosa* Infection in Cystic Fibrosis: Pathophysiological Mechanisms and Therapeutic Approaches. Expert Rev. Respir. Med..

[33] Semenyuk E.G., Laning M.L., Foley J., Johnston P.F., Knight K.L., Gerding D.N., Driks A. (2014). Spore Formation and Toxin Production in *Clostridium difficile* Biofilms. PLoS ONE.

[34] Crowther G.S., Chilton C.H., Todhunter S.L., Nicholson S., Freeman J., Baines S.D., Wilcox M.H. (2014). Comparison of Planktonic and Biofilm-Associated Communities of *Clostridium difficile* and Indigenous Gut Microbiota in a Triple-Stage Chemostat Gut Model. J. Antimicrob. Chemother..

[35] Doan T.-H.-D., Yen-Nicolaÿ S., Bernet-Camard M.-F., Martin-Verstraete I., Péchiné S. (2020). Impact of Subinhibitory Concentrations of Metronidazole on Proteome of *Clostridioides difficile* Strains with Different Levels of Susceptibility. PLoS ONE.

[36] Pantaléon V., Soavelomandroso A.P., Bouttier S., Briandet R., Roxas B., Chu M., Collignon A., Janoir C., Vedantam G., Candela T. (2015). The *Clostridium difficile* Protease Cwp84 Modulates Both Biofilm Formation and Cell-Surface Properties. PLoS ONE.

[37] Liao S., Bitoun J., Nguyen A., Bozner D., Yao X., Wen Z.T. (2015). Deficiency of PdxR in *Streptococcus mutans* Affects Vitamin B6 Metabolism, Acid Tolerance Response and Biofilm Formation. Mol. Oral Microbiol..

[38] Soavelomandroso A.P., Gaudin F., Hoys S., Nicolas V., Vedantam G., Janoir C., Bouttier S. (2017). Biofilm Structures in a Mono-Associated Mouse Model of *Clostridium difficile* Infection. Front. Microbiol..

[39] Pantaléon V., Monot M., Eckert C., Hoys S., Collignon A., Janoir C., Candela T. (2018). *Clostridium difficile* Forms Variable Biofilms on Abiotic Surface. Anaerobe.

[40] Purcell E.B., McKee R.W., McBride S.M., Waters C.M., Tamayo R. (2012). Cyclic Diguanylate Inversely Regulates Motility and Aggregation in *Clostridium difficile*. J. Bacteriol..

[41] Metcalf D., Sharif S., Weese J.S. (2010). Evaluation of Candidate Reference Genes in *Clostridium difficile* for Gene Expression Normalization. Anaerobe.

[42] Denève C., Deloménie C., Barc M.-C., Collignon A., Janoir C. (2008). Antibiotics Involved in *Clostridium difficile*-Associated Disease Increase Colonization Factor Gene Expression. J. Med. Microbiol..

[43] Chen X., Katchar K., Goldsmith J.D., Nanthakumar N., Cheknis A., Gerding D.N., Kelly C.P. (2008). A Mouse Model of *Clostridium difficile*—Associated Disease. Gastroenterology.

[44] Péchiné S., Janoir C., Boureau H., Gleizes A., Tsapis N., Hoys S., Fattal E., Collignon A. (2007). Diminished Intestinal Colonization by *Clostridium difficile* and Immune Response in Mice after Mucosal Immunization with Surface Proteins of Clostridium Difficile. Vaccine.

