# Supplementary material for: Impact of Subinhibitory Concentrations of Metronidazole on Morphology, Motility, Biofilm Formation and Colonization of Clostridioides difficile
Source: Antibiotics (Basel). 2022 May 5;11(5):624. doi: 10.3390/antibiotics11050624 (PMC9137534; doi:10.3390/antibiotics11050624)
Supplement: Supplementary file 1 [file antibiotics-11-00624-s001.zip › antibiotics-1675016-supplementary-done.pdf]

antibiotics

MDPI

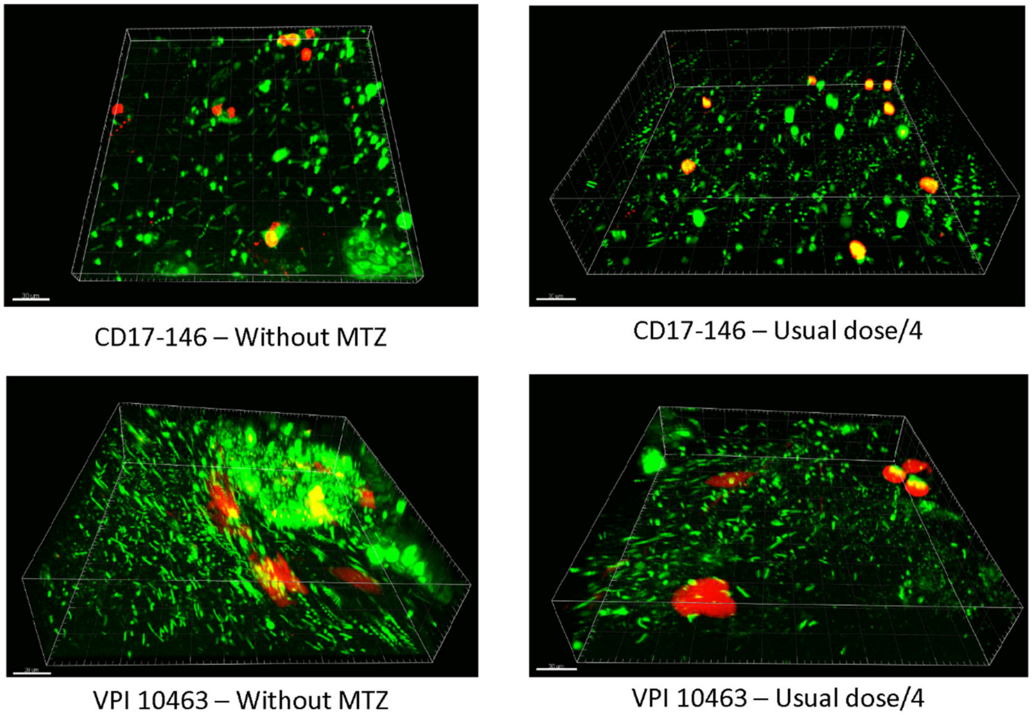

Figure S1: Heterogeneous distribution of *C. difficile* over the cecal tissue in a mono-associated mouse model. Confocal laser-scanning microscopy 3-D projection of tissue-associated bacteria obtained from cecum for the CD17-146 without or with treatment of MTZ at 0.125 mg/kg, and the VPI 10463 without or with treatment of MTZ 0.125 mg/kg. Live cells (bacterial [rod] or epithelial) are labeled in green, dead cells are labeled in red. Scale bars (white): 30 µm.

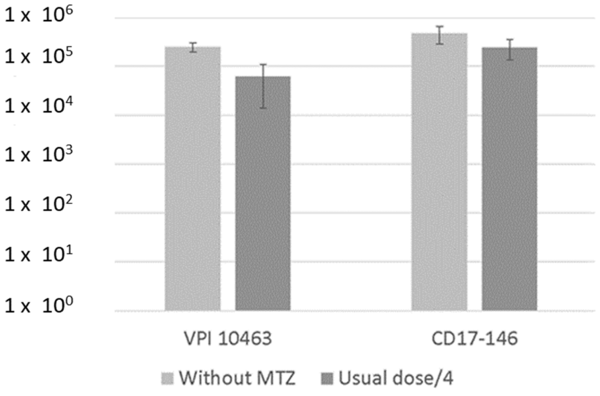

Figure S2. Impact of MTZ on cecum colonization of *C. difficile* in monoxenic mice, 7 days post-infection. Germ-free mice were infected by either VPI 10463 (group A and B) or CD17-146 (group C and D) strain with 5 × 10^5 CFU of *C. difficile*. From 1-day post-infection, mice were treated with sterile water (group A and C) or with a quart of usual dose of MTZ: 12.5 mg/kg (group B and D) for 7 days by oral gavage twice a day. *C. difficile* shedding was monitored in feces at day 7 post-infection. There were no significant differences in colonization between the group treated with MTZ at usual dose/4 and the group non-treated for both strains. The error bars represent standard error of the mean (SEM).

Table S1 : Sequences of oligonucleotide primers used in this study

Name	Sequence (5' to 3')
flgBF	GCAACTAATCTAAGAAGTCAGACAATAGC
flgBR	AGGCATAGCATCATTTAGTGTTTCTTC
fliAF	GAATATGCCTCTTGTAAAGAGTATAGCA
fliAR	TGCATCAATCAATCCAATGACTCC
gluDF	CAGTAGGGCCAACAAAAGGT
gluDR	TCCACCTTTACCTCCACCAT
fliC146F	TTAACACAATTAAAAGATGAGATTGAAAGA
fliC146R	AAACATTAGTTCCATAACTCTCCAACTG
fliCVPIF	GAATCAAGAATAAGAGATACAGATGTTG
fliCVPIR	ATAATTGTAAAACTCCTTGTGGTTGTTG
